# Gamma-oryzanol rich fraction regulates the expression of antioxidant and oxidative stress related genes in stressed rat's liver

**DOI:** 10.1186/1743-7075-7-23

**Published:** 2010-03-24

**Authors:** Maznah Ismail, Ghanya Al-Naqeeb, Wan Abd Aziz bin Mamat, Zalinah Ahmad

**Affiliations:** 1 Nutrigenomics Programme, Laboratory of Molecular Biomedicine, Institute of Bioscience, Universiti Putra Malaysia, 43400 UPM Serdang, Selangor Darul Ehsan, Malaysia; 2 Faculty of Medicine and Health Sciences, Universiti Putra Malaysia, 43400 UPM Serdang, Selangor Darul Ehsan, Malaysia

## Abstract

**Background:**

Gamma-oryzanol (OR), a phytosteryl ferulate mixture extracted from rice bran oil, has a wide spectrum of biological activities in particular, it has antioxidant properties.

**Methods:**

The regulatory effect of gamma-oryzanol rich fraction (ORF) extracted and fractionated from rice bran using supercritical fluid extraction (SFE) in comparison with commercially available OR on 14 antioxidant and oxidative stress related genes was determined in rat liver. Rats were subjected to a swimming exercise program for 10 weeks to induce stress and were further treated with either ORF at 125, 250 and 500 mg/kg or OR at 100 mg/kg in emulsion forms for the last 5 weeks of the swimming program being carried out. The GenomeLab Genetic Analysis System (GeXPS) was used to study the multiplex gene expression of the selected genes.

**Results:**

Upon comparison of RNA expression levels between the stressed and untreated group (PC) and the unstressed and untreated group (NC), seven genes were found to be down-regulated, while seven genes were up-regulated in PC group compared to NC group. Further treatment of stressed rats with ORF at different doses and OR resulted in up-regulation of 10 genes and down regulation of four genes compared to the PC group.

**Conclusions:**

Gamma-oryzanol rich fraction showed potential antioxidant activity greater than OR in the regulation of antioxidants and oxidative stress gene markers.

## Background

Rice bran is a rich natural source of vitamin E, containing up to 300 mg/kg [1]. It possesses 3000 mg/kg of gamma-oryzanol (OR), which is a mixture of 10 ferulate esters of triterpene alcohol [2,3]. OR has been reported to contribute to multiple health beneficial activities, including, reduction of cholesterol levels [4], inhibition of platelet aggregation [5] and antioxidant functions [6].

Supercritical fluid extraction (SFE) of lipid has received attention as an alternative method to organic solvent extraction and has been shown to be an ideal method for extracting and fractioning oils [7]. Supercritical CO2 is non-toxic, non-flammable, and simple to use when compared to conventional organic solvents. Furthermore, SFE fractionation allows the pool of target compounds in the oil fraction. These advantages may make supercritical carbon dioxide extraction ideal in the food and pharmaceutical industries [8]. Previous study by Xu and Godber [2], has demonstrated that SFE produces high oil yield and able to concentrate OR compared to solvent extraction in rice bran oil. However, only a few publications on SFE fractionation of rice bran oil to produce bioactive rich fractions are available until now. In this study, SFE was employed to extract and fractionate OR in rice bran as gamma-oryzanol rich fraction (ORF) which also contains other antioxidant molecules such as tocopherols, tocotrienols and ferulates.

Physical exercises are generally recognized to have a positive impact on physiological parameters affecting overall health [9]. Even though there are many known health benefits of exercise, there is strong evidence suggesting that strenuous exercise may cause oxidative stress in both animals and human studies [10,11]. Previous studies have shown that exercise at high intensity can increase the generation of reactive oxygen species (ROS) in liver and skeletal muscle which leads to oxidative stress [12]. Several studies involving physical exercises have been conducted using laboratory animals, especially rats. Swimming in small laboratory animals has been widely used for studying the physiological changes and the capacity of the organism in response to stress [13]. A positive aspect of this training method lies in the ability of rats to swim [14]. Protection against ROS and the breakdown products of oxidized lipids and proteins is provided by antioxidant enzymes such as catalase (CAT), superoxide dismutase (SOD) and glutathione peroxidase (GPX). In recent years, studies have been intensively performed on supplementation of natural antioxidant compounds to attenuate oxidative stress-induced pathogenesis of diseases [15]. However some of the antioxidant molecules are labile to degradation in the presence of oxygen, water and light, or are not absorbed well. Hence it becomes all the more appropriate to use a delivery system which will augment their stability and hence enhance the performance.

An effective approach for achieving efficient supplementation delivery would be to rationally develop nanosystems based on the understanding of the specific supplementation active compound interactions with the biological environment targeted [16]. For the last 2 decades, nanosystems with different composition and biological properties have been extensively investigated for drug, gene [17] and supplement delivery application. A variety of nanoparticles of different structural and chemical formulations have been tested for their target-specificity and as drug carrier systems. Numerous scientific research works have been performed to test the use of magnetic nanoparticles in the treatment of carcinogenic brain tumour cells and breast cancer cells; colloid gold nanoparticles, liposomes and polymeric micelles as drug delivery systems to target tumour cells and deliver anticarcinogenic drug in a controlled manner [18]. Drug delivery systems (DDS) based on the enhanced permeability and retention (ERF) effect has been explored for better therapeutic approach. In this regard, nanoparticles hold tremendous potential as an effective drug delivery system. For therapeutic applications, supplement can either be integrated in the matrix of the particle or attached to the particle surface. Another example of drug delivery aspect of nanomedicine is the use of nanomaterials including peptide-based nanotubes to target the vascular endothelial growth factor (VEGF) receptor and cell adhesion molecules as a control measure of disease progression [16]. Gene expression analysis is used to analyze the function of one or more genes. Single gene analysis is not practical for medium to high-throughput applications in terms of the amount of time, labor and cost required to process the samples. In a research that requires a moderately large number of genes to be assayed, a medium to high-throughput method is needed. Thus, quantitative analysis of multiplexed genes expression in a single reaction, from a limited amount of total RNA, is of great use to research scientists. In this study, the GenomeLab™ Genetic Analysis System GeXP (Beckman Coulter Inc. USA) was used to study the multiplex gene expression of 14 antioxidant and oxidative stress related genes. The rats were put initially on a 10 weeks exercise swimming program to induce stress and followed by treatment with ORF and OR for the last 5 weeks.

## Materials and methods

### Rice bran samples

Rice bran samples were obtained from local milling company, National Rice Board Sdn (Bernas) at Kuala Selangor, (Malaysia). Samples were stabilized and stored at 4°C before extraction process was being carried out.

### Chemicals

Gamma-oryzanol, triolein and tween 80 were purchased from Sigma (Sigma-Aldrich Co., St. Louis, Missouri). Methanol, acetonitrile and dichloro methane (HPLC grade), (Fisher Scientific Co Ltd., Ottawa, ON). RiboPure™ RNA isolation kit (Ambion, Austin, TX, USA). GeXP starting kit, PCR and reverse transcription kit were purchased from Beckman Coulter (Beckman Coulter Inc. USA).

### Preparation of gamma-oryzanol rich fraction ORF

Gamma-oryzanol rich fraction was prepared from stabilized rice bran using supercritical fluid extractor (SFE) (Thar 1000 F, Thar Technologies, Inc., Pittsburgh, PA, USA). One hundred g of the dried samples were pulverised for 3 min in a stainless steel grinder (Waring Commercial, Torrington, CT, USA) and placed into a one liter stainless steel SFE extraction vessel. Extraction procedures were set at pressure of 600 bars and temperature of 40°C. The pressure within the extraction vessel was generated with a constant carbon dioxide flow rate at 30 g/min and regulated by an automated back pressure regulator. The extraction process lasted for 3 h and ORF was collected from the collection vessel after depressurization of the SFE system. Fractionantion was done in order to produce rice bran oil with higher oryzanol content. The method for the fractionantion process is similar to the extraction process. However, in fractionaon process, range of pressure at 100 bar - 300 bar and temperature at 40°C - 60°C were applied in the the first separator in order to get the optimun condition for fractionantion. The ORF produced using SFE parameters according to the procedure above is rich in OR (2.6 ± 0.17% w/w) in comparison to OR content in Rice bran oil (0.46 ± 0.01% w/w), which is extracted by conventional Soxhlet procedure (unpublished data).

### Animal study

#### Preparation of ORF and OR emulsions

Both ORF and OR were administrated to the rats orally in the emulsion form. ORF at various dosage was slowly added to 20 ml distilled water and 1% tween 80. Emulsions were prepared at room temperature (25°C) using a laboratory scale homogenizer (Ultra-turax T25 basic, IKA^®^-WERKE GmbH & Co. KG, Staufen, Germany) at 13000 rpm for 5 min. OR emulsion was prepared by dissolving calculated amount of OR in 1 ml of triolein and prepared according to the same procedure as ORF emulsion. Rats were fed daily 2 ml of the freshly prepared emulsion in the morning by gavage.

#### Animal groups

Male Sprague-Dawley rats weighing 250 - 300 g were purchased from As-Sapphire Sdn Bhd (Selangor, Malaysia). The animals were fed standard rat pellet (As-Sapphire, Selangor, Malaysia) and tap water. They were housed at 28 ± 2°C on a 12 hours dark and 12 h light cycle. All procedures were approved by the Animal Care and Use Committee, Faculty of Medicine and Health Sciences, Universiti Putra Malaysia. Six experimental rat groups were established (6 rats per each group) as follows: group 1, unstressed and untreated (NC) group were put into the shallow water with no treatment given, group 2, stressed and untreated group (PC) rats were subjected to exercise swimming program for 10 weeks without any treatment given, group 3, OR group, rats were subjected exercise swimming program for 10 weeks with treatment of 100 mg/kg OR emulsion daily for the last 5 weeks and groups 4 to 6, ORF emulsion groups (ORFL, ORFM and ORH) rats were subjected to exercise swimming program for 10 weeks with administration of ORF emulsion at 125 mg/kg, 250 mg/kg and 500 mg/kg respectively, for the last 5 weeks.

#### Swimming exercise program

Rats of the same strain, sex and weight, were trained to swim 60 min/day, 5 days a week, during 10 weeks, in the same device where the swimming trials took place. Exercise sessions lasted 10 min on the first day of the training period and were increased by 10 min, each 7 days. At the end of the 7th day the animals swam continuously for 20 min and at the end of the 14th day, they swam for 40 min. Continuous exercise for 60 min was performed from the 28^th ^day until the end of the training period. Unstressed and untreated rats (NC) placed in shallow water at 31 ± 2°C, 5 days/week, were used as controls. The amount of food taken by rats was recorded once a week, while the body weights were recorded every 2 weeks using weighing machine (AND, HR-200, Singapore). At the end of the experiment, all rats were dissected and liver tissues for RNA isolation were removed, snap frozen in liquid nitrogen and immediately stored at -80°C.

#### RNA isolation

RNA was isolated from frozen liver samples using the RiboPure™ RNA Isolation Kit (Ambion, Austin, TX, US) according to the manufacturer's instructions.

#### Primer design

Primers were designed using GenomeLab eXpress Profiler software. Fragment sizes ranged from 150 to 350 nt with a 7-nucleotide minimum separation size between each PCR product. Genes and primer sequences are listed in Table 1 and 2. In addition to the 14 genes of interest, each panel contained an internal control gene (Kanr) and three normalization genes (Actb, Gapdhs and 18S). Reverse primers which consisted of 20 nucleotides complementary to the target gene were tagged to a 19-nucleotide universal reverse sequence. Forward primers consisted of 20 nucleotides corresponding to the target gene were tagged to a 18 nucleotides universal forward sequence. All primers were synthesized by Proligo (France SAS) and supplied by Sigma Aldrich from the gene sequence of rat (*rattus norvegicus*) which was adopted from the NCBI (National Center for Biotechnology Information) GenBank Database http://www.ncbi.nlm.nih.gov. GeXPS primer stocks were diluted in nuclease-free water to a final concentration of 500 nM for reverse primer sets, and diluted to a final concentration of 200 nM for forward primer.

**Table 1:** Gene name, gene locus and gene product used in GeXP multiplex analysis of antioxidant and oxidative stress related genes in rat liver

Gene Name	Gene Locus	Gene Product/Description	Function
Ubb	NM_138895	Ubiquitin B	Mediates ATP-dependent degradation (stress response-related gene)
18S ^a^	BC168964	18S	Housekeeping genes
Nfkbib	NM_030867	Nuclear factor of kappa light polypeptide	Stress response-related gene
Akr1b1	NM_012498	Aldo-keto reductase family 1, member B1	Stress response-related gene
Grm5	NM_017012	Glutamate receptor, metabotropic5	Stress response-related gene
Apo E	NM_138828	Apolipoprotein E	Lipid metabolism(stress response-related gene)
Stip1	NM_138911	Stress-induced phosphoprotein 1	Stress response-related gene
Cox11	NM_001109575	COX11 homolog, cytochrome c oxidase	Oxidation
(Mt1a)	NM_138826	Metallothionein 1a	Stress response-related gene
Oxsr1	NM_001108194	Oxidative-stress responsive 1	Stress response-related gene
GPX	NM_183403	Glutathione peroxidase 2	Antioxidant
Gapdhs ^a^	NM_023964	Glyceraldehyde-3-phosphate dehydrogenase	Housekeeping genes
SOD1	NM_017050	Superoxide dismutase 1,	Antioxidant
Hao1	NM_001107780	Hydroxyacid oxidase 1, liver	Stress response-related gene
NADH	NM_001130505	NADH dehydrogenase	Nuclear gene encoding mitochondrial protein (stress response-related gene)
Actb ^a^	NM_031144	actin, beta	Housekeeping genes
CAT	NM_012520	catalase	Antioxidant

Knar ^b^			Internal control

^a ^Gene used for normalization

^b ^Internal control

**Table 2:** Gene name, gene product size, and forward and reverse primer sequences used in GeXP assays of antioxidant and oxidative stress related genes in rat liver

Gene Name	Fragment Size	Left Sequence w/Universals	Right Sequence w/Universals
Ubb	187	AGGTGACACTATAGAATAGACACCATCGAGAACGTGAA	GTACGACTCACTATAGGGAGACAAGGTGCAGGGTTGACT
18S ^a^	194	AGGTGACACTATAGAATAGCTCCAGGACGGAGTTCATA	GTACGACTCACTATAGGGACAGCAGGTGGAGCTCTGATT
Nfkbib	204	AGGTGACACTATAGAATACCCGAGGATGAGGATGATAA	GTACGACTCACTATAGGGATCATCAGGAAGAGGTTTGGC
Akr1b1	210	AGGTGACACTATAGAATACGCAGAAGTCTGAAGCTGTG	GTACGACTCACTATAGGGACTGGTACTGCCCTCCACATT
Grm5	213	AGGTGACACTATAGAATAGCCAACTTTAATGAGGCCAA	GTACGACTCACTATAGGGATGATGTACACCTTCGGGACA
Apo E	230	AGGTGACACTATAGAATAGAAGATGAAGGCTCTGTGGG	GTACGACTCACTATAGGGACTCTGCAGCTCTTCCTGGAC
Stip1	236	AGGTGACACTATAGAATAACTACAACAAATGCCGGGAG	GTACGACTCACTATAGGGATGGCACTTCTTGAGCACATC
Cox11	244	AGGTGACACTATAGAATACTTCCTTCCCCCATCTGTTT	GTACGACTCACTATAGGGACTGTTCCTCACAATGGCTCA
(Mt1a)	250	AGGTGACACTATAGAATACACCAGATCTCGGAATGGAC	GTACGACTCACTATAGGGAACTGTTCGTCACTTCAGGCA
Oxsr1	257	AGGTGACACTATAGAATACAGCGATTGAACTAGCCACA	GTACGACTCACTATAGGGATTGTGCCTCAACAGTTCTGC
GPX	271	AGGTGACACTATAGAATATCAACATCGAGCCTGACATC	GTACGACTCACTATAGGGACAGACTTAGAGCCCCCAGTG
Gapdh ^a^	279	AGGTGACACTATAGAATAATCAATGGATTTGGACGCAT	GTACGACTCACTATAGGGAAGCTCCAGGGGATTTCCTTA
SOD1	285	AGGTGACACTATAGAATACTTGCTTTTTGCTCTCCCAG	GTACGACTCACTATAGGGAAAAATGAGGTCCTGCAGTGG
Hao1	293	AGGTGACACTATAGAATACCTGTCAGACCATGGGAACT	GTACGACTCACTATAGGGATGAGCTGTGGTGGTAGCTTG
NADH	298	AGGTGACACTATAGAATAGTGAAGCCCATTTTCAGTCG	GTACGACTCACTATAGGGATAATGTGTGTCCGCTGCTTC
Actb ^a^	307	AGGTGACACTATAGAATAATGTACGTAGCCATCCAGGC	GTACGACTCACTATAGGGAAGGGCAACATAGCACAGCTT
Cat	314	AGGTGACACTATAGAATAGTGGTTTTCACCGACGAGAT	GTACGACTCACTATAGGGACACGAGGTCCCAGTTACCAT
Knar ^b^	325	AGGTGACACTATAGAATAATCATCAGCATTGCATTCGATTCCTGTTTG	GTACGACTCACTATAGGGAATTCCGACTCGTCCAACATC

^a ^Gene used for normalization

^b ^Internal control

#### cDNA synthesis

50 ng of RNA from each sample was reverse transcribed with multiplex universal reverse primers. The reverse transcription reactions were performed according to GenomeLab™ GeXP Start Kit from Beckman Coulter protocol. The RT reaction was performed in a thermal-cycler with the following program: 48°C for 1 min; 37°C for 5 min; 42°C for 60 min; 95°C for 5 min and hold at 4°C.

#### PCR amplification

Subsequently, PCR was done with each reaction mixture containing 9.3 μl of the cDNA from each of the above reverse transcription reaction product, and 2 μl of 200 nM forward universal primer set mix, 4 μl 25 mM MgCl_2_, 0.7 μl of Thermo Start Taq DNA polymerase (Thermo Fisher Scientific, Pittsburgh, PA) and 4 μl of 5× PCR Master Mix buffer (GenomeLab GeXP Start Kit; Beckman Coulter, inc). Amplification conditions consisted of initial denaturation at 95°C for 10 min, followed by 35 two-step cycles of 94°C for 30 sec and 55°C for 30 sec, ending in a single extension cycle of 68°C for 1 min. The reactions were performed in a XP Thermal Cyclers (BIOER; Technology, Germany).

### GeXP multiplex data analysis

PCR products from multiplex primer reactions were diluted 2: 8 in water, and l μl of this solution was added to 38.5 μl sample loading solution along with 0.5 DNA size standard 400 (GenomeLab GeXP Start Kit; Beckman Coulter, Inc). The GeXPS system is used to separate PCR products based on size by capillary gel electrophoresis and to measure their dye signal strength in arbitrary units (A.U.) of optical fluorescence, defined as the fluorescent signal minus background.

### Fragment analysis and gene expression signature analysis

The data were initially analyzed using the Fragment Analysis module of the GeXP system software. Then, data were imported into the analysis module of eXpress Profiler software. Actb, Gapdhs and 18S genes were tested for result consistency. As 18S gene gave consistent results it was chosen for normalizing all the data for all the interested genes.

### Statistical analysis

ANOVA and Duncan grouping were performed by using SPSS window program version 14.0 to identify significant differences between groups (P < 0.05).

## Results

**Figure 1:**
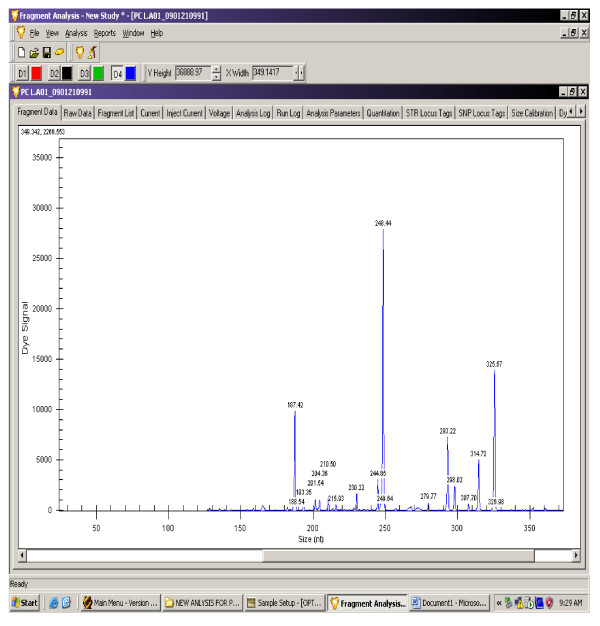
**A representative electropherogram from the GeXP multiplex analysis in NC group**.

**Figure 2:**
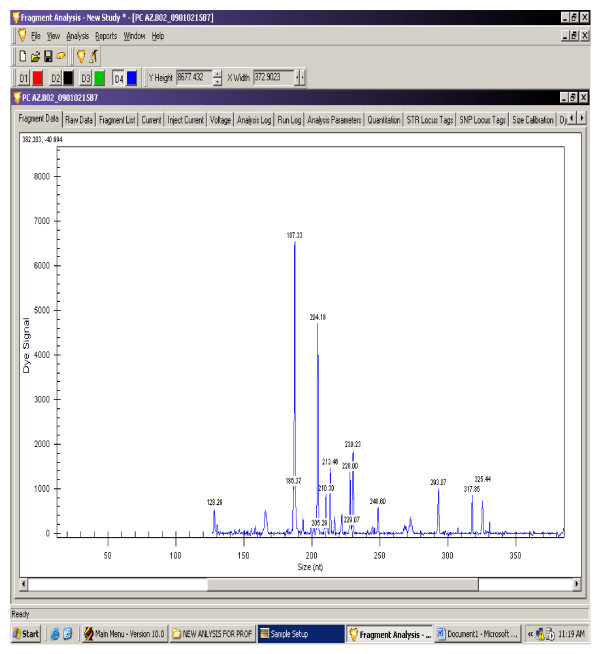
**A representative electropherogram from the GeXP multiplex analysis PC group**.

**Figure 3:**
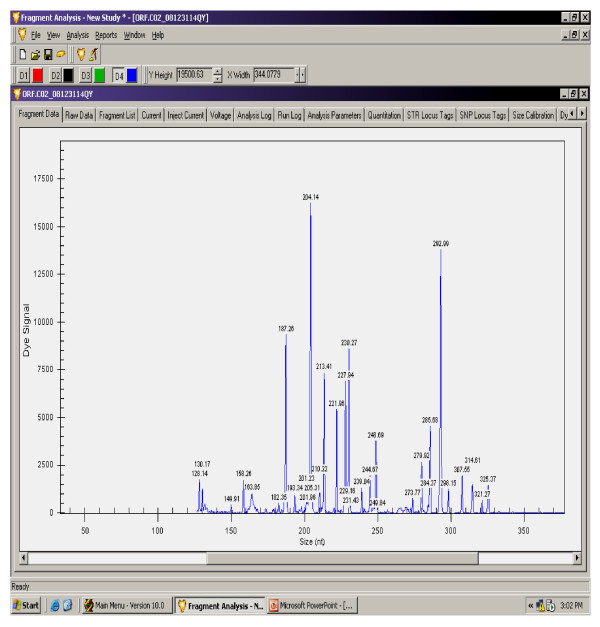
**A representative electropherogram from the GeXP multiplex analysis of gamma-oryzanol rich fraction at 125 mg/kg (ORFL) treated group**.

**Figure 4:**
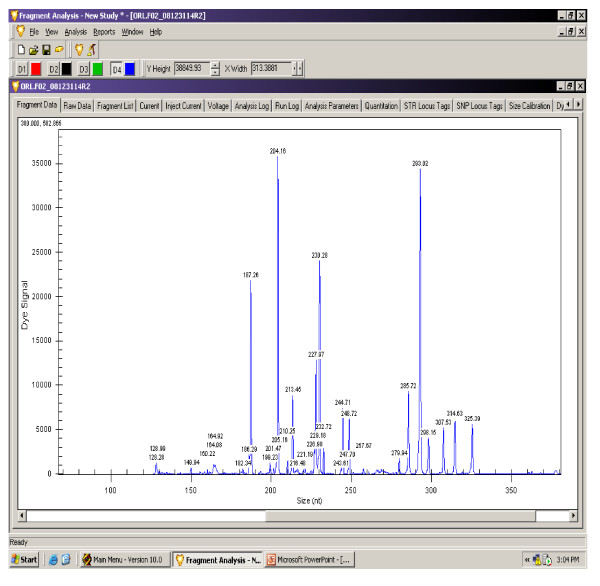
**A representative electropherogram from the GeXP multiplex analysis of gamma-oryzanol rich fraction at 250 mg/kg (ORFM) treated group**.

**Figure 5:**
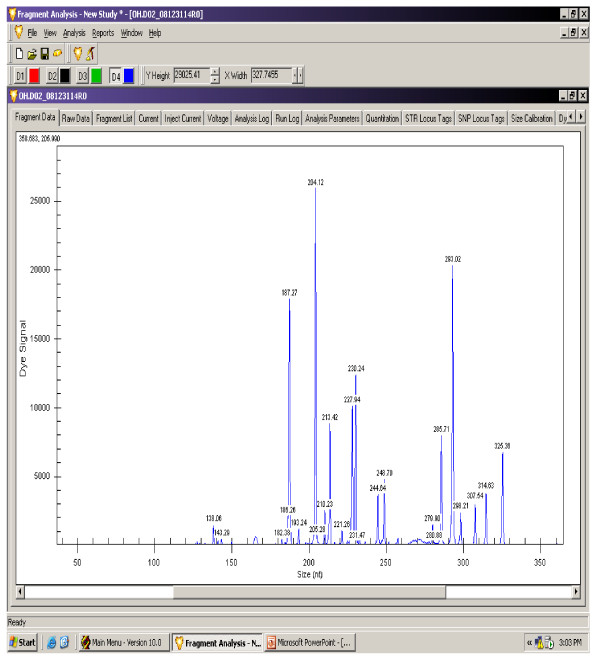
**A representative electropherogram from the GeXP multiplex analysis of gamma-oryzanol rich fraction at 500 mg/kg (ORFH) treated group**.

**Figure 6:**
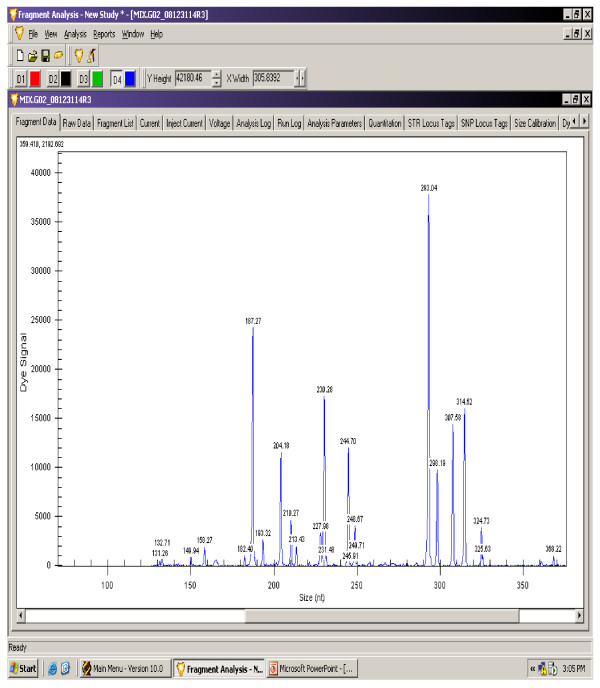
**A representative electropherogram from the GeXP multiplex analysis of gamma-oryzanol at 100 mg/kg (OR) treated group**.

**Figure 7:**
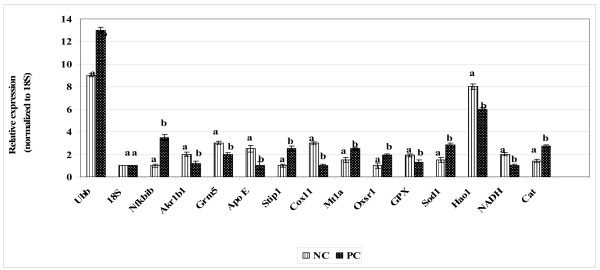
**Relative expression of 14 antioxidants and oxidative stress related genes in stressed and untreated rats (PC) and unstressed untreated rats (NC)**. Each value represents mean of 3 rats ± SD. Data was normalized with 18S gene. Within each gene Different alphabets indicate significant difference (*P *< 0.05).

The gene expression levels of 14 antioxidant and oxidative stress related genes were monitored using multiplex GeXP analysis system. The electropherogram initial data by fragment analysis from the 14 genes multiplex assay are shown in Figure 1, Figure 2, Figure 3, Figure 4, Figure 5, Figure 6. The Knar peak at 325 nucleotides size serves as an internal control for the multiplex. As shown in Figure 7, out of 14 genes, seven genes in PC group were down-regulated while, seven genes were up-regulated significantly compared to NC group. The ubiquitin B (Ubb) gene was found to be highly expressed in PC group compared to NC group, whereas, hydroxyacid oxidase 1 and liver (Hao1) gene were found to be highly suppressed in PC group compared to NC group followed by metallothionein 1 (Mt1a) gene. Genes related to antioxidative process including SOD1 and CAT were up-regulated significantly in PC group compared to NC group. Whereas, GPX was down-regulated significantly in PC group compared to NC group.

**Figure 8:**
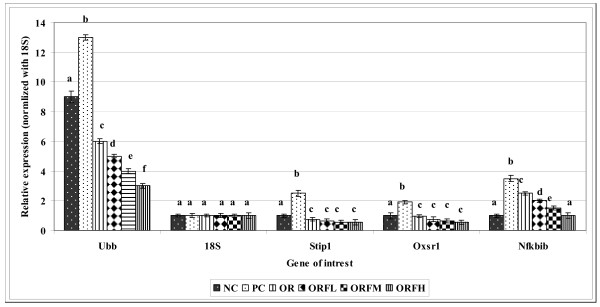
**Relative suppression of 14 antioxidants and oxidative stress related genes treated with OR and ORF**. NC = unstressed and untreated rats, PC = stressed and untreated rats OR = group treated with gamma-oryzanol at 100 mg/kg, ORFL = group treated with gamma-oryzanol rich fraction at 125 mg/kg, ORFM = group treated with gamma-oryzanol rich fraction at 250 mg/kg, ORFH = group treated with gamma-oryzanol rich fraction at 500 mg/kg. Each value represents means of 3 rats ± SD. Within each gene different alphabets indicate significant difference (*P *< 0.05).

As shown in Figure 8, further treatment of stressed rats with either ORF emulsion at different doses or OR emulsion had resulted in down-regulation of ubiquitin B (Ubb), stress-induced phosphoprotein 1 (Stip1), nuclear factor of kappa light polypeptide (Nfkbib) and oxidative-stress responsive 1 (Oxsr1) genes significantly (P < 0.05) compared to PC group. ORF treated groups showed higher suppression level of Ubb and Nfkbib genes compared to OR treated group. Among different doses of ORF emulsions, it was observed that the suppression level of Ubb and Nfkbib genes was concentration dependent, whereby higher expression level was obtained when higher dose of ORF was applied compared to PC group. Among the treated groups, there was no significant different in the suppression level of Stip1 and Oxsr1 genes. Furthermore, different doses of ORF showed no significant differences in the suppression level of Stip1 and Oxsr1 genes.

**Figure 9:**
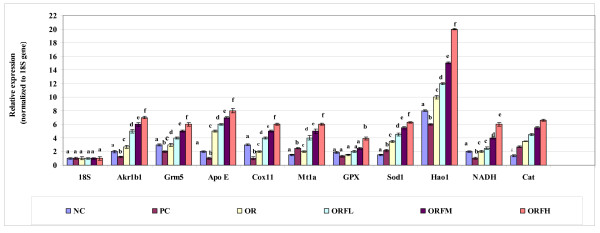
**Relative expression of 14 antioxidants and oxidative stress related genes by OR and ORF treatment**. NC = unstressed and untreated rats, PC = stressed and untreated rats OR = group treated with gamma-oryzanol at 100 mg/kg, ORFL = group treated with gamma-oryzanol rich fraction at 125 mg/kg, ORFM = group treated with gamma-oryzanol rich fraction at 250 mg/kg, ORFH = group treated with gamma-oryzanol rich fraction at 500 mg/kg. Each value represents means of 3 rats ± SD. Within each gene different alphabets indicate significant difference (*P *< 0.05).

As shown in Figure 9, further treatment of rats with either ORF or OR resulted in up-regulation of 10 genes compared to the PC group. The most significant gene expression responses to ORF and OR treatment observed was hydroxyacid oxidase 1, liver (Hao1), followed by Apo E gene and genes related to antioxidant including SOD1 and CAT. As response to stress, both glutamate receptor, metabotropic 5 (Grm5) and Aldo-keto reductase family 1, member B1 (Akr1b1) mRNA were down in PC group compared to NC group. Further treatments with ORF and OR resulted in up-regulation of Grm5 and Akr1b1 significantly compared to PC group. Nevertheless, the expression level of the up-regulated genes (Figure 9) in ORF treated groups at different doses showed to be higher compared to control groups (PC and NC) and OR treated rats in a dose-dependent manner. This explains that oral administrations of ORF at different doses are capable in up-regulating the tested genes. This indicates that, ORF contains other bioactive compounds besides OR, which might contribute synergistically to the regulating of the above antioxidants and oxidative stress related genes.

## Discussion

In this study the regulatory effect of ORF extracted and fractionated from rice bran oil using SFE with high concentration of OR in comparison with commercially available OR were studied on 14 genes related to antioxidant and oxidative stress response. Throughout this experiment, 600 bar and 40°C were chosen as the SFE parameters for extraction the ORF. The idea of using bioactive rich fraction as ORF is to compare whether its activity is higher than its counterpart pure compound, henceforth making it a more attractive nutraceutical. In this study, both ORF and OR were administrated to the rats in emulsion form which allows an increase in absorption of lipophilic compounds [19].

We have chosen swimming because it is a natural behaviour of rats [20,21] and can prevent foot injury, causing less impact and a reduced degree of muscle trauma [22]. In our study, stress response-related genes inculding Ubb, 1 Stip1, Nfkbib and Oxsr1 were up-regulated in PC group compared to NC group. Together, the up-regulation of the above stress response-related genes observed in the PC group indicates that this experimental group achieved a good level of fitness indicating, the success of the swimming exercise program as oxidative stress inducer. Oxidative stress has been shown to induce the activity of ubiquitin [23]. Stress-induced phosphoprotein 1 (STIP1) protein and Oxsr1 were also reported to be enhanced in response to oxidative stress [24].

Pervious studies have reported that oxidative stresses can also induce Nfkbib activation in HeLa cells [25]. The inhibition of Nfkbib activation by a variety of antioxidants and by over expression of antioxidant enzymes has been reported [26]. At molecular level we are reporting here, that oxidative stress induced by swimming to the rats increased the expression of activated Nfkbib mRNA level by 3.5 fold in PC group compared to NC group. The down-regulation of Ubb, Stip1, Nfkbib and Oxsr1 genes by ORF emulsion at different doses and OR explained the molecular mechanism of the antioxidant activity of ORF and OR.

In the present study, we have shown that hepatic Hao1 and Apo E mRNA levels were down-regulated in PC group compared to NC group that was due to oxidative stress induced by swimming. On the other hand, treatment with ORF emulsion at different doses or gamma-oryzanol emulsion caused up-regulation of this gene. Oxidative stress has been shown to reduce the Hao1 mRNA expression [27]. Previous study reported by Espiritu *et al*. [28], showed that oxidant stress in 3T3-L1 cells and adipose tissue from lean mice significantly reduced Apo E mRNA level. The down-regulation of Apo E by oxidative stress might be due to activation of Nfkbib transcription, and its effect on Apo E [29]. On the other hand, both ORF and OR treatments enhanced the expression of Apo E mRNA level significantly compared to PC group.

Metallothionein (Mt1) is considered as general stress proteins, and its transcription has been shown to be affected by oxidative stress [30]. In this study, Mt1 gene was up-regulated in PC group compared to NC group in response to oxidative stress. Further treatment with ORF or OR resulted in up-regulation of Mt1 mRNA level significantly compared to PC group. As mentioned previously, Mt1 acts as a scavenger of reactive oxygen species in cultures of cells isolated from mice (deficient in both Mt1) [31]. In response to the stress, glutamate receptor, metabotropic 5 (Grm5), mRNA was down regulated. Previous study was shown that the activation of Grm5 in HT-22 cells and rat cortical neuron cultures protects cells from glutamate toxicity and other forms of stress [32]. The up-regulation of Grm5 by GORF and OR treated groups in concentration depend manner, might decreased the extracellular glutamate content which enhance reactive oxygen species formation [33].

Our study also showed that swimming rats for 10 weeks increased the mRNA level of CAT and SOD in PC group compared to the NC group. On the other hand, the mRNA level of GPX was decreased in PC group. Previous study reported by Gore et al. [34], has shown that the mRNA levels of SOD1 and CAT were not altered by exercise, may be was due to different type of exercise applied. However, our results are in agreement with those reported by Fridovich [35], in which exercise decreased GPX mRNA levels compared to control rats. The up-regulation of SOD1, CAT and GPX mRNA levels significantly in treated groups with GORF and OR compared to PC group explain the antioxidant properties of ORF and OR. Oryzanol and vitamin E in rice bran have reported significant antioxidant activities which protect cells from oxidative damage of plasma very low-density lipoprotein, cellular proteins and DNA [2].

Although OR content in ORF (26 mg/g) is lower than the concentration administrated in GOR group (100 mg OR), the up-regulation and down-regulation of tested genes in ORF group exhibited greater antioxidant activity in comparison to OR group. Results from our findings clearly reveal that ORF contains other bioactive compounds, which may contribute to the regulation of tested genes. Nevertheless, OR in ORF is one of the major bioactive compounds that contributes to antioxidative improvement and regulation of antioxidant and oxidative stress related genes of stressed rat liver. The antioxidant activity of OR has been reported in the literature [6].

Tocopherols and tocotrienols are other main antioxidants present in the rice bran [36]. They may contribute independently or synergistically with OR for the improvement of antioxidant capacity and regulation of the tested genes. Beside tocopherols and tocotrienols, rice bran oil was reported to be rich in the phenolic compounds (2.51-3.59 mg/g) and phytosterols (0.5%) [37], and oleic acid (38.4%) [36], which may contribute directly to antioxidative action [38,39].

Since both ORF and OR were administrated to the rats in emulsion form, new developments of materials should be carried out to achieve an effective delivery system. Applications of nanoparticles are widespread, ranging from confined reaction vessels to drug carriers or shells protecting enzymes. The use of nanoparticles in drug delivery systems gives rise to several advantages such as higher drug loading with smaller dose volume, site-specific sustained drug delivery, faster absorption of bioactive compounds and improved patient discomfort owing to the reduced dimension of such drug delivery system. Development of techniques, such as soft lithography, that can be cheaply and easily used to fabricate micro and nano devices without the need for microfabrication facilities, has greatly enhanced the widespread application of microscale technologies in drug discovery [40] and delivery techniques. Successful drug delivery will have enormous academic, clinical and practical impacts on gene therapy, cell and molecular biology, pharmaceutical and food industries, and bio-production [41]. Therefore, novel methods that can improve the predictability of the performance of drugs in the body can be useful in minimizing the high costs associated with finding and validating new drugs efficiency.

Hosseinkhania *et al*. [42], have approved that the conjugation of dextran derivatives with chelate residues based on metal coordination is a promising way to enable plasmid DNA to target the tumor in gene expression as well as to prolong the duration of gene expression. Konishia, *et al*. [43], has reported that dual sustained release of cisplatin (CDDP) and adriamycin (ADM) from a biodegradable hydrogel attached to the tumor synergistically enhanced their in vivo anti-tumor effect through the trans-tissue delivery. As new developments of materials to achieve an effective drug delivery system, the hydrophobically modified glycol chitosan (HGC) was self-assembled to DNA nanoparticles for efficient gene transfers. The HGC nanoparticles were proven to have effectively delivery of DNA to COS-1 cells in the presence of serum. Animal study also confirmed that HGC nanoparticles could be used as a potent gene delivery vehicle in vivo [44]. The complexation with poly (ethylene glycol) (PEG) -engrafted cationized dextran in combination with ultrasound (US) irradiation is a promising way to target the NK4 plasmid DNA to the tumor for gene expression [45]. Therefore, developments of ORF nanparticles are underway in our laboratory to for achieving efficient supplementation delivery.

## Conclusion

Our findings indicate that ORF up-regulates the antioxidant genes while down-regulates the oxidative stress genes marker, possibly due to the presence of many potent antioxidants.

## Abbreviations

ORF: Gamma-oryzanol rich fraction; OR: Gamma-oryzanol; SFE: supercritical fluid extraction; GeXP: GenomeLab Genetic Analysis System; NC: untreated normal control; PC: exercised untreated rats; ORFL: exercised treated rats with gamma-oryzanol rich fraction at 125 mg/kg; ORFM: exercised treated rats with gamma-oryzanol rich fraction at 250 mg/kg; ORFH: exercised treated rats with gamma-oryzanol rich fraction at 500 mg/kg; OR: exercised treated rats with gamma-oryzanol at 100 mg/kg.

## Competing interests

The authors declare that they have no competing interests.

## Authors' contributions

All authors were involved in the design of this study; and performed laboratory analyses and statistics. The manuscript was written by all the authors.
